# Closed-Loop digital therapeutics empowered by deep reinforcement learning and wearable sensing for precision orthopedic rehabilitation: a simulation-based proof-of-concept study

**DOI:** 10.3389/fresc.2026.1822939

**Published:** 2026-06-11

**Authors:** Jiahao Dong, Tao Chen, Zhongyu Peng

**Affiliations:** 1 First Clinical Medical College, Yunnan University of Traditional Chinese Medicine, Kunming, China; 2 Yunnan Provincial Hospital of Chinese Medicine, Kunming, China

**Keywords:** deep reinforcement learning, wearable sensing, prognostic prediction, patient-generated health data, orthopedic rehabilitation, personalized medicine, intelligent rehabilitation system, temporal convolutional network

## Abstract

**Objective:**

Static, one-size-fits-all protocols in postoperative orthopedic rehabilitation fail to adapt to individual recovery dynamics, potentially leading to suboptimal rehabilitation efficiency or an elevated risk of secondary injury. To address this critical gap, we propose and provide a simulation-based proof-of-concept validation for a novel closed-loop management system that deeply integrates patient-generated health data (PGHD) with deep reinforcement learning (DRL), offering a potential technical pathway for real-time personalized optimization of rehabilitation regimens and continuous prediction of long-term functional outcomes.

**Methods:**

A three-tier system architecture was constructed, comprising an intelligent sensing layer, an AI decision-making and prediction layer, and an interactive feedback layer. Through wearable inertial measurement units (IMUs) and surface electromyography (sEMG) devices, the system continuously collected multi-dimensional PGHD, including movement quality, training intensity, adherence, and pain feedback. These heterogeneous data were encoded into a comprehensive “patient state space” through a standardized feature engineering pipeline. A proximal policy optimization (PPO) algorithm was employed to train a DRL agent to learn the optimal policy for dynamically adjusting the next-cycle rehabilitation prescription (including exercise type, intensity, frequency, and progression pace). The agent aimed to maximize a hierarchical cumulative reward function that integrates short-term safety (*Δ*VAS pain score monitoring), mid-term adherence (training completion rate), and long-term functional improvement. Critically, a temporal convolutional network (TCN) prognostic module was deeply coupled with the DRL agent, providing prospective predictions of future functional recovery curves that inform the agent's long-term reward calculation, equipping the system with the capability of “making decisions based on predictions.”

**Results:**

A proof-of-concept validation was conducted in a simulated environment for a post-operative anterior cruciate ligament reconstruction (ACLR) scenario. The trained DRL agent demonstrated the ability to generate differentiated rehabilitation strategies: in stratified analysis, it prescribed distinct progression paces for virtual patients with fast vs. slow recovery trajectories. Under this simulation setting, compared with a static conservative protocol, the DRL-driven strategy reduced the simulated time to “safe return to light activity” by an average of 15% and, compared with a static aggressive protocol, relatively decreased simulated “re-injury” events by 40%. The mean absolute errors (MAEs) of the TCN prognostic module for predicting functional scores at 2, 4, and 8 weeks into the future were 3.2, 4.8, and 6.5 points (on a 100-point scale), respectively, outperforming the ARIMA, LSTM, and GRU baseline models. An ablation study confirmed the TCN module's independent contribution, as its removal led to a relative increase in the simulated re-injury rate.

**Conclusion:**

This proof-of-concept study provides foundational evidence for the technical feasibility of a DRL-based closed-loop rehabilitation system. The proposed framework uniquely couples a wearable sensing layer with a symbiotic DRL-TCN architecture, demonstrating the potential to safely and dynamically personalize rehabilitation strategies in a simulated environment. These findings lay the groundwork for future prospective clinical trials, which are the necessary next step to validate safety, efficacy, and clinical utility in real-world settings.

## 1 Introduction

Post-operative orthopedic rehabilitation management is progressively evolving from a standardized, static paradigm toward a personalized and dynamic approach. This trend is both an intrinsic requirement of advancing clinical practice and a benefit of the continuous development of digital health technologies. Among these, wearable sensing devices, characterized by their non-invasive and continuous monitoring capabilities, have become crucial tools for achieving objective quantification and real-time assessment of the rehabilitation process. By collecting multi-modal physiological data such as gait, joint range of motion, and electromyographic signals in real time, wearable systems can provide dynamic information beyond traditional scales, offering data-driven support for rehabilitation decisions (1). In scenarios involving orthopedic degenerative diseases, post-traumatic conditions, and spinal cord injuries, wearable devices with embedded sensor networks enable the quantitative assessment of gait symmetry, joint range of motion, and muscle activation patterns, with data acquisition accuracy reaching over 90% of that of clinical electromyography, thus providing an objective basis for formulating rehabilitation plans (2). For instance, a randomized controlled trial (RCT) on knee osteoarthritis (KOA) demonstrated that a wearable device utilizing an IMU significantly improved patients' Lysholm scores and reduced inflammatory factor levels (3). Technologies such as IMUs and sEMG have been widely applied in post-operative rehabilitation for anterior cruciate ligament reconstruction and total knee arthroplasty, showing good validity in quantifying motor function and identifying abnormal patterns, though they still face challenges such as signal interference and individual variability. Clinical validation studies require the establishment of a standardized evaluation framework, including verification of IMU kinematic parameter accuracy through synchronized video-based motion analysis systems, validation of sEMG signal quality against the gold standard of needle electromyography, and confirmation of clinical predictive value through large-sample RCTs (4). For example, a tai chi rehabilitation protocol customized based on IMU/sEMG data significantly improved gait speed and motor function in stroke patients in an RCT (5). Nevertheless, a fundamental limitation persists: the majority of current rehabilitation protocols still rely on static, periodic clinical assessments, which suffer from irreducible feedback delays and an inherent inability to adapt prescriptions in real time to a patient's dynamically changing recovery status.

In recent years, artificial intelligence, particularly deep reinforcement learning (DRL), has shown potential in medical decision support. By constructing a closed-loop optimization mechanism of “state-action-reward,” DRL can adapt to complex, continuous decision-making environments and has made initial progress in areas such as chronic disease management and rehabilitation robot control. For example, in the anti-infective treatment of sepsis, a DRL model guided by clinical knowledge effectively improved the prognostic outcomes of antibiotic recommendation protocols (6); in intermittent androgen deprivation therapy for prostate cancer, a DRL framework also enabled dynamic, personalized adjustment of dosing strategies (7). However, translating this potential to multi-variable, heavily constrained scenarios like orthopedic rehabilitation is non-trivial. Key challenges include high data heterogeneity across patients, insufficient model interpretability—often referred to as the “black box” problem—and, most critically, difficulties in establishing rigorous safety boundaries to prevent injury during the learning and execution of policies (8–10).

Concurrently, patient-generated health data (PGHD) provides a new data foundation for continuous and dynamic rehabilitation modeling. PGHD encompasses multi-dimensional information—including physiological parameters, behavioral logs, and subjective symptom reports—continuously collected by patients or wearable devices. It can supplement the limitations of traditional electronic health records in temporal continuity and contextual completeness, thereby supporting more precise individualized interventions (11, 12). Nonetheless, the standardization, quality control, and privacy protection of PGHD remain critical issues that must be addressed during its clinical translation.

Therefore, to bridge this gap and explore potential technical pathways for the intelligent evolution of post-operative orthopedic rehabilitation, this original research article aims to propose and rigorously validate, within a simulation-based environment, a novel closed-loop system framework that deeply integrates PGHD and DRL for dynamic rehabilitation optimization and prognostic prediction.

The system continuously monitors patient status (Monitoring) through an intelligent sensing layer (wearable IMU/sEMG and patient reports). Multi-modal PGHD is transmitted to the cloud-based AI core, where a Temporal Convolutional Network (TCN) prognostic module predicts future functional recovery. This output is utilized in the long-term reward calculation of the Deep Reinforcement Learning (DRL) agent to generate a personalized rehabilitation prescription (Prediction & Decision). The prescription is delivered to the patient-end App for execution (Intervention) via an interactive feedback layer, while clinicians can review all AI suggestions through a monitoring dashboard, ensuring safety and clinical control (Human-in-the-Loop). The entire system forms a dynamically optimized intelligent management closed loop.

**Figure 1:**
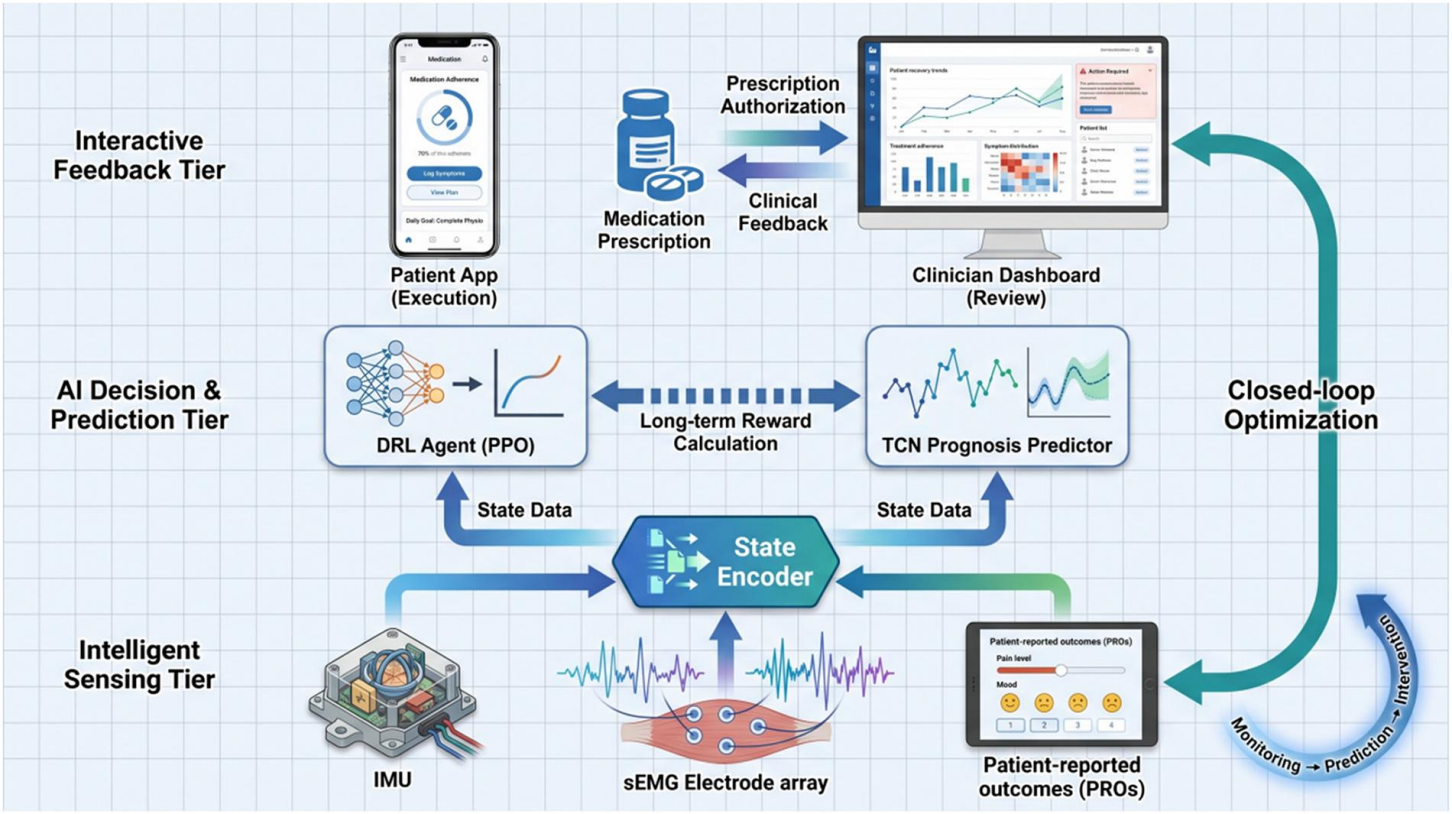
Framework of the PGHD- and DRL-based closed-loop intelligent management system for post-operative orthopedic rehabilitation.

As illustrated in Figure 1, the system constructs an intelligent management closed loop of “monitoring-prediction-intervention.” It continuously collects multi-modal patient data through the intelligent sensing layer. In the cloud-based AI core, the DRL agent and the TCN prognostic module work in concert to generate personalized rehabilitation prescriptions and predictions. The interactive feedback layer ensures that the clinician retains the final decision-making authority. This study aims to provide a rigorous proof-of-concept validation of the system framework's technical feasibility through comprehensive simulation modeling and analysis, offering methodological evidence and prospective references for subsequent clinical translation research.

## 2 Methods

### 2.1 Overall system architecture and clinical workflow integration

The proposed system employs a modular three-tier architecture, meticulously designed to align with the clinical rehabilitation pathway and achieve seamless closed-loop management from data acquisition to interventional feedback.

Intelligent Sensing Layer (Data Acquisition End):

This layer is responsible for the non-invasive, continuous collection of multi-modal physiological and behavioral data, including kinematic data from IMUs (e.g., 3-axis accelerometer and gyroscope data sampled at 100 Hz), electromyographic activity data from sEMG, and patient-reported outcomes (PROs) collected via a mobile application. All devices synchronize and transmit data in real time using Bluetooth Low Energy (BLE).

AI Decision-Making and Prediction Layer (Cloud Processing Center):

This layer constitutes the intelligent core of the system, containing a state encoder, the DRL agent, a prognostic prediction module, and a safety monitoring module. It receives data from the sensing layer, outputs personalized rehabilitation prescription recommendations and prognostic predictions, and ensures decision safety.

Interactive Feedback Layer (User End):

This layer translates AI decisions into executable instructions and visual feedback, including a patient-facing rehabilitation guidance App and a clinician-facing monitoring and review dashboard, ensuring human-in-the-loop clinical control.

### 2.2 Data standardization and clinical state space construction

**Figure 2:**
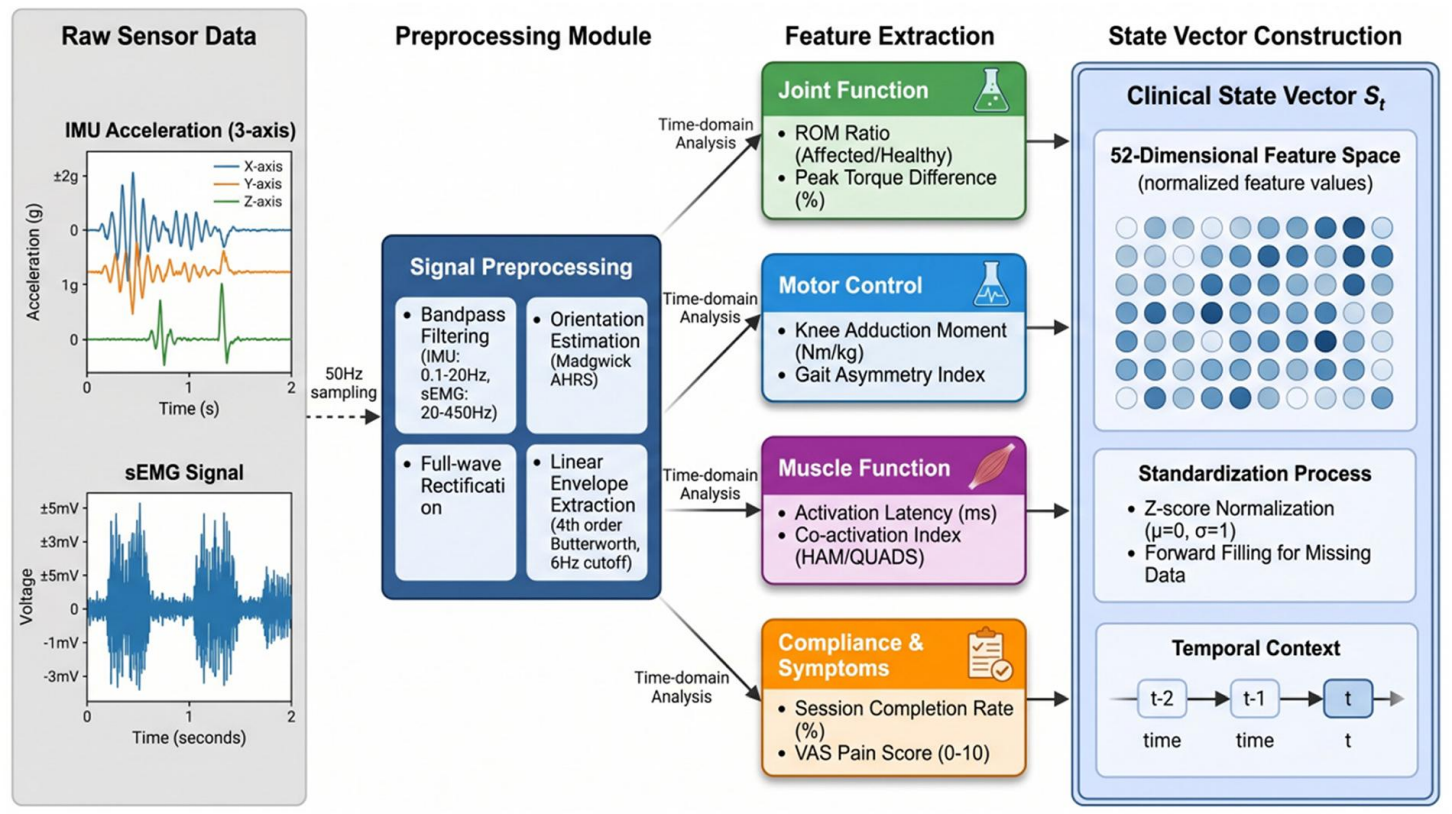
Flowchart for constructing the patient clinical state space from multi-modal sensor data.

To transform raw, heterogeneous sensor data into structured information suitable for model learning, we established a standardized feature engineering pipeline (Figure 2). This pipeline aims to extract features with explicit clinical significance from multi-modal PGHD and construct a comprehensive “patient state space.”

Raw IMU kinematic signals and sEMG electromyographic signals undergo standardized pre-processing (e.g., filtering, attitude calculation). Subsequently, four major categories of clinical features are extracted from the pre-processed signals: joint function, motor control, muscle function, and adherence & symptoms. These dynamic features, along with static baseline features (demographics, surgical information) and temporal contextual features (post-operative day), are collectively encoded into a fixed-dimensional numerical vector, forming the “patient state space St” perceived by the DRL agent.

To achieve model generalizability, we established rigorous data preprocessing and feature engineering protocols, with all extracted features referencing and integrating validated methods from existing research. Traditional kinematic assessment primarily relies on IMUs or optical motion capture systems; while they can accurately record macroscopic kinematic parameters like joint angles and displacements, they struggle to directly reflect neuromuscular control mechanisms (13). In contrast, sEMG technology quantifies microscopic physiological characteristics such as motor unit recruitment patterns and muscle co-activation timing by detecting electrical activity signals from muscles. The combination of these two modalities forms a comprehensive assessment framework spanning from biomechanics to neurophysiology (14).

Raw Signal Processing:

IMU data underwent attitude calculation using a complementary filter. sEMG signals were processed through band-pass filtering (20–500 Hz), full-wave rectification, and smoothing to extract the linear envelope of muscle activation, a standard preprocessing method for analyzing muscle function. The standardized pipeline for sEMG signal processing is a key component for ensuring data reliability and research reproducibility, encompassing three main stages: signal acquisition, pre-processing, and feature extraction/analysis.

Clinical Feature Extraction: For each assessment cycle (24 h), the following core clinical indicators were calculated:

Joint Function Category:

Ratio of active/passive range of motion between the affected and unaffected sides; peak torque difference in simulated isokinetic strength testing.

Motor Control Category:

Peak knee adduction moment and sway path length of the center of gravity during standardized tasks (e.g., sit-to-stand transfer); asymmetry index of spatiotemporal parameters in the gait cycle. These biomechanical features have been demonstrated to be highly correlated with functional recovery after knee injuries. The clinical translational value of gait analysis is further underscored by its utility as a screening tool. Traditional PRO assessments can be influenced by patients' subjective cognitive biases, whereas gait parameters (such as the peak moment in the early stance phase) can objectively identify high-risk individuals requiring intensified intervention (15).

Muscle Function Category: Activation delay time of target muscles; co-activation index of antagonist muscles.

Adherence and Symptom Category: Training completion rate; average subjective pain VAS score.

State Space (St) Definition:

The above dynamic features were collectively encoded with static baseline features (demographics, surgical information) and temporal contextual features (post-operative day) into a fixed-dimensional numerical vector, forming a comprehensive “clinical portrait” of the patient at time *t*. All continuous features were normalized using Z-score standardization. For sporadic missing data caused by improper device wear or transmission errors, a forward-filling method was applied. The final state vector dimension was *d = 52*.

### 2.3 Clinically-Oriented design of the deep reinforcement learning model

**Figure 3:**
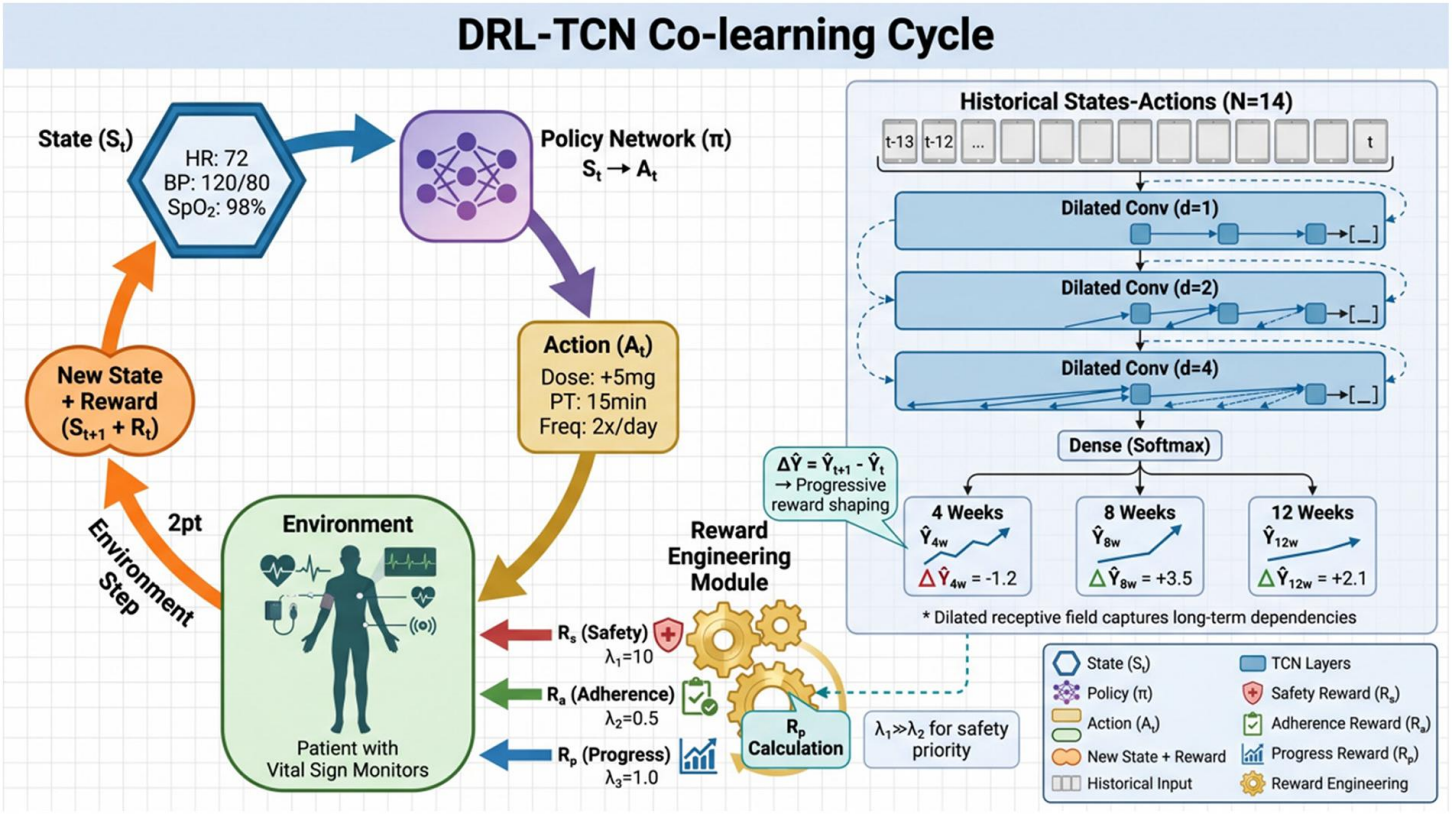
Collaborative working mechanism of the DRL agent and TCN prognostic prediction module.

The DRL framework was designed as a “virtual rehabilitation therapist.” We employed the Proximal Policy Optimization (PPO) algorithm to construct the agent, which is deeply coupled with an independent TCN prognostic prediction module to jointly enable prospective decision-making (Figure 3). This algorithm was chosen for its advantages in training stability and sample efficiency, which have been successfully applied in medical scenarios requiring continuous, safe decision-making, such as blood glucose regulation and mechanical ventilation parameter optimization. For instance, by designing an “opt-out” default framework, the PPO policy increased the accuracy of medication dosage calculations for obese patients by 37%–39% while avoiding the alert fatigue commonly associated with traditional clinical decision support systems (16). This “nudge” mechanism innovatively balances clinical autonomy with best-practice guidance, with its theoretical basis lying in the conservative nature of PPO policy updates, which maintains the stability of decision boundaries (17).

Action Space (At) Design:

Corresponding to the adjustment of the prescription for the next rehabilitation cycle (e.g., 72 h), it is a hybrid space tailored to clinical habits. The specific definitions are as follows:

Training Modality Selection (Discrete Action ad): Comprising 4 options: {Add balance training, Add strength training, Maintain current regimen, Reduce weight-bearing}.

Training Dosage Adjustment (Continuous Action ac): A scaling factor for the current training intensity, with a range of [−0.5, 0.5].

Progression Decision (Discrete Action ap): Whether to introduce a higher-difficulty exercise from the next phase, comprising 2 options: {Yes, No}.

To implement this hybrid action space, the output end of the PPO policy network adopts a multi-head structure: two heads output the logits for discrete actions, and one head outputs the mean and log standard deviation for the continuous action.

Reward Function (Rt) Design:

A hierarchical weighted design was adopted to guide the agent in learning policies that align with clinical objectives. The weights for each reward component were calibrated through a structured, two-stage process. First, a grid search over weight combinations was conducted on a smaller virtual cohort of 200 patients to identify Pareto-optimal trade-offs among safety, adherence, and progress. Second, these candidate weightings were presented to two senior orthopedic surgeons, whose structured feedback was used to select the final configuration that best reflected clinical priorities—specifically, the principle that a safety violation should dominate the cumulative reward.

Safety Reward (Rs):

A large negative reward (*Rs = −1.0*) is applied if the average VAS pain score increase within a single assessment cycle *Δ*VAS > 2, or if an abnormal movement pattern (defined as a gait asymmetry index exceeding a preset threshold of 20%) is detected; otherwise, it is 0.

Adherence Reward (Ra): Positively correlated with the training completion rate ct, defined as Ra = ct.

Functional Progress Reward (Rp):

Linked to the improvement in objective functional indicators and the long-term functional gain predicted by the prognostic module. Specifically defined as *Rp = (Lysholmt - Lysholmt-1)/10 + *α* * ΔŶt + 4w*, where Lysholm is the functional score of the current cycle, *ΔŶt + 4w* is the change in functional score predicted by the TCN for the next 4 weeks, and *α* is a balancing coefficient set to 0.5.

Total Reward: *Rt = *λ*1 * Rs + *λ*2 * Ra + *λ*3 * Rp*. The final weights were set as *λ1 = 10.0, *λ*2 = 0.5, *λ*3 = 1.0*, ensuring that a single safety violation (Rs = −1.0) incurs a penalty of −10.0, an order of magnitude larger than any positive progress reward achievable in a single cycle. This clinically-driven trade-off prioritizes “do no harm” above all else.

Algorithm Implementation: The policy network (π) takes the state St as input and outputs the action probability distribution, striking a balance between exploration and exploitation.

The DRL agent (left) observes the current patient state St and outputs a rehabilitation prescription action At via its policy network π. The action is executed in the environment (patient/simulation model), generating a new state *St + 1* and an immediate reward Rt. The reward function integrates three dimensions: safety (Rs), adherence (Ra), and functional progress (Rp). Crucially, the functional progress reward Rp receives a key input from the TCN prognostic module's (right) prediction of the future functional recovery curve Ŷ. The TCN analyzes historical state-action sequences through a dilated convolutional architecture, and its prediction enables the DRL to assess the long-term value of current decisions, thus achieving prospective optimization.

### 2.4 Prognostic prediction module: temporal modeling

To provide prospective decision support, the system integrated an independent prognostic prediction model. We employed a Temporal Convolutional Network (TCN), whose dilated causal convolution structure can effectively capture long-range dependencies with high computational efficiency. TCNs have demonstrated superior performance over traditional models like recurrent neural networks (RNNs) in medical time-series tasks such as ECG classification and disease progression prediction, particularly in handling long sequences and mitigating vanishing gradients. As an emerging deep learning architecture, it has recently shown significant advantages in medical time-series data analysis. Its core value lies in the efficient capture of both short- and long-term dependencies, parallel computing capability, and adaptability to irregular temporal patterns. Compared to traditional RNNs or Long Short-Term Memory (LSTM) networks, TCNs, through dilated convolutions and multi-layer stacking structures, can model complex temporal dynamics in long sequences at a lower computational cost (18, 19).

Input and Output: The input is a sequence of historical states and actions from the past *N = 14* cycles, and the output is the standard functional score (e.g., Lysholm score) for the future *T = 4, 8, 12* weeks.

Co-Training Paradigm:

The TCN module and the DRL agent were trained using an iterative collaborative approach. First, the TCN was pre-trained using initial sequence data generated by the DRL's random exploration in the simulation environment. Subsequently, during the DRL training iterations, after a certain number of policy updates, the TCN was fine-tuned using the newly generated interaction data. In this process, the DRL relied on the TCN's predictions to calculate the long-term functional gain term *Δ*Ŷ in the reward function, while the TCN learned more robust “intervention-response” dynamics from the diverse policy trajectories explored by the DRL.

### 2.5 Simulation-Based proof-of-concept and safety assessment

Prior to clinical introduction, we conducted a proof-of-concept validation in a highly controllable simulation environment. This approach draws on the research paradigm in computational biomechanics and rehabilitation engineering, where simulation is used to pre-emptively evaluate the safety of interventional strategies. For example, by coupling macroscopic skeletal muscle dynamics with microscopic myofiber electrophysiological properties, the temporal relationship between muscle fatigue and strength recovery during rehabilitation training can be more accurately predicted (20, 21). Furthermore, interactive simulation platforms based on virtual reality (VR) allow rehabilitation physicians to adjust training parameters (e.g., resistance magnitude or range of motion) in real-time and provide haptic feedback via force-feedback devices, thereby compensating for the lack of physical realism in traditional VR (22, 23).

Simulation Environment Construction:

We developed a simulation environment driven by physiology and post-operative ACLR knowledge, with the following core components and assumptions:

Lower Limb Musculoskeletal Biomechanical Model:

A simplified two-dimensional sagittal plane model was adopted, encompassing major muscle groups such as the quadriceps and hamstrings. The muscle force-length-velocity relationship was based on a simplified Hill muscle model, and joint moments were estimated via inverse dynamics.

Tissue Healing Temporal Function: Graft healing strength was modeled as a sigmoid function of post-operative time *H(t) = H_0_ + (H_max_ - H_0_)/(1 + e^-k(t-t_0_^^)^)*, with parameters set based on animal model healing literature.

“Load-Response” Relationship Model: If the load borne by the tissue exceeds the current healing strength H(t) by a safety factor (set at 1.2), a “re-injury” simulation event is triggered, leading to a sharp decline in the functional score and a reset of the healing timeline. The load level is determined by the DRL's actions.

Virtual Patient Behavioral Model: Incorporated individual differences, including “recovery potential” (affecting the healing speed parameter *k*), “adherence” (affecting the deviation between actual executed dose and prescribed dose), and “pain sensitivity” (affecting the mapping between physiological load and VAS report). Each virtual patient was parameterized by sampling *k* from a log-normal distribution [*μ* = ln(0.15), *σ* = 0.3] to represent the spectrum of “fast” to “slow” recovery phenotypes. Adherence was sampled from a truncated normal distribution with a mean of 0.80 and a standard deviation of 0.15 [range: (0.5, 1.0)], while pain sensitivity was drawn from a uniform distribution [0.8, 1.2] acting as a multiplicative factor on the standard load-to-VAS mapping.

Formal Definition of “Safe Return to Light Activity":

In the simulation, this endpoint was defined as: for two consecutive assessment cycles, the affected limb's quadriceps strength reaching ≥70% of the unaffected side, and a gait asymmetry index <10% during the completion of a standard “sit-to-stand” transfer task.

Training and Testing:

The DRL agent was trained in an environment of 1,000 virtual patients and subsequently tested head-to-head against two predefined static protocols in a cohort of 500 new virtual patients, independently and identically distributed from the training set. The conservative static protocol mandated a fixed 10% increase in training load per week with no new modalities introduced before week 8, while the aggressive static protocol applied a 25% weekly load increase and introduced advanced maneuvers at week 4, irrespective of patient state. These rule-based protocols were chosen to represent the extremes of current clinical practice. Key indicators, including simulated time-to-return and simulated re-injury rate, were evaluated, along with the prognostic prediction module's accuracy.

### 2.6 Simulation and training details for reproducibility

To ensure full reproducibility of our results, we provide here a comprehensive account of the computational environment, model architectures, and hyperparameter configurations used in this study.

Computational Environment and Implementation: All models were implemented in Python 3.9 using PyTorch 2.0. The PPO agent was built using the Stable-Baselines3 library (v2.1.0). Training and testing were conducted on a workstation equipped with an Intel Core i9–13900 K CPU, 64 GB of RAM, and an NVIDIA GeForce RTX 4090 GPU.

PPO Hyperparameters: The PPO agent was configured with the following hyperparameters, tuned through a preliminary grid search on a smaller validation cohort: learning rate, 3e-4; discount factor (*γ*), 0.99; generalized advantage estimation (GAE) lambda (*λ*), 0.95; clip range, 0.2; batch size, 64; number of epochs per update, 10; entropy coefficient, 0.01; and value function coefficient, 0.5. The policy network and value network shared a common backbone consisting of 3 fully connected layers of 256 units each with ReLU activation functions, followed by separate output heads for the policy and value.

TCN Architecture: The TCN comprised 4 residual blocks with exponentially increasing dilation rates (1, 2, 4, 8), each containing two dilated causal convolutional layers with 128 filters of kernel size 3. Weight normalization and dropout (rate=0.1) were applied after each convolution. The model was trained using the Adam optimizer with a learning rate of 1e-3 and a mean squared error (MSE) loss, for a maximum of 500 epochs with early stopping (patience=25 epochs). The total number of trainable parameters for the TCN was 1,247,000.

Training Procedure: The DRL agent was trained for a total of 2 million environment steps across 1,000 virtual patients. After every 100,000 steps, the policy was evaluated on a held-out validation set of 100 patients to monitor for overfitting. The TCN was pre-trained on 500,000 sequences collected from the DRL agent's random policy and was subsequently fine-tuned every 500,000 steps during DRL training. The complete training process took approximately 18 h on the hardware described above.

## 3 Results

**Figure 4:**
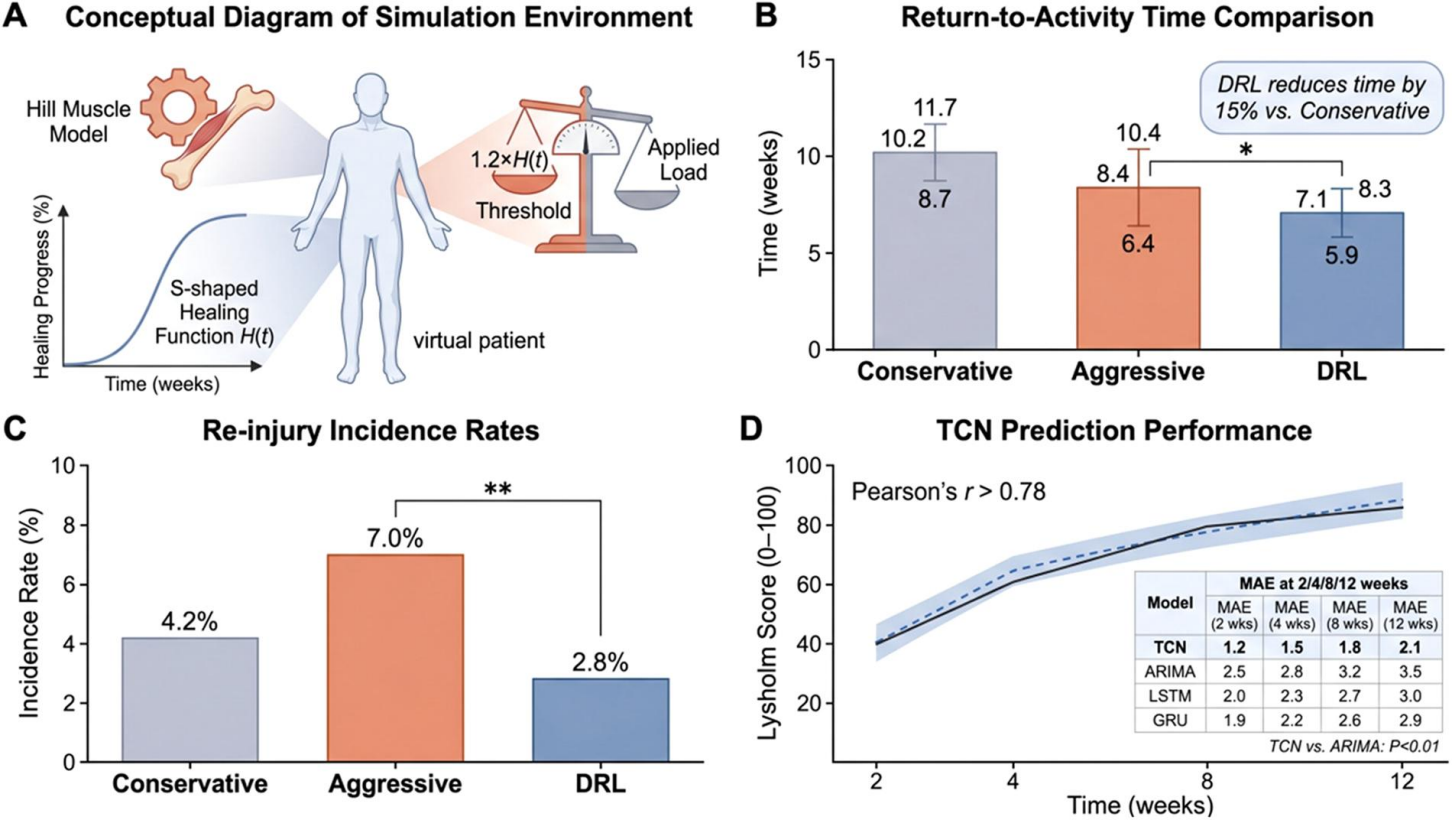
Proof-of-concept results from simulation-based validation. **(A)** Conceptual diagram of the simulation experiment environment, including the virtual patient model and biomechanical simulation. **(B)** Comparison of the time required to achieve the ‘safe return to light activity' standard among the three rehabilitation protocols. The DRL protocol required statistically less time than the static conservative protocol (*P* < 0.05). **(C)** Incidence of ‘re-injury' events during simulated rehabilitation among the three protocols. The DRL protocol’s incidence was significantly lower than that of the static aggressive protocol (**P < 0.001). **(D)** Prediction curves from the TCN prognostic prediction module vs. actual values for functional scores at 2, 4, 8, and 12 weeks into the future; the inset table shows the Mean Absolute Error (MAE) at each time point.

In a simulated post-ACLR environment comprising 1,000 virtual patients, the proposed DRL closed-loop system was trained and tested. Under this simulation setting, the system demonstrated potential advantages in simulated rehabilitation efficiency, safety, and prognostic prediction accuracy compared to static rehabilitation protocols (Figure 4). A detailed analysis follows.

(A) Conceptual diagram of the simulation experiment environment, including the virtual patient model and biomechanical simulation. (B) Comparison of the time required to achieve the “safe return to light activity” standard among the three rehabilitation protocols. The DRL protocol required statistically less time than the static conservative protocol (*P* < 0.05). (C) Incidence of “re-injury” events during simulated rehabilitation among the three protocols. The DRL protocol's incidence was significantly lower than that of the static aggressive protocol (**P < 0.001). (D) Prediction curves from the TCN prognostic prediction module vs. actual values for functional scores at 2, 4, 8, and 12 weeks into the future; the inset table shows the Mean Absolute Error (MAE) at each time point.

### 3.1 Clinical pattern analysis of DRL agent's learned policy

After training, the DRL agent demonstrated highly adaptive decision-making capabilities in the simulation. *post-hoc* analysis of the learned policy revealed structural similarities between its decision patterns and the general principles of rehabilitation therapy. This similarity indicates that when the reward function is meticulously designed to encode core clinical objectives, the DRL agent can autonomously induce decision rules from data that partially align with expert experience. Research has shown that in the Kellgren-Lawrence (KL) grading task for knee osteoarthritis (KOA), AI assistance improved the assessment accuracy of junior physicians from 0.76 ± 0.018 to 0.86 ± 0.013 (*P* < 0.001) and significantly enhanced inter-rater agreement (24). Similarly, in radiographic hip fracture detection, the diagnostic performance (F1 score 0.942) of an AI algorithm based on multi-model voting approached that of senior orthopedic physicians (0.938) (25). Specifically, in this study, the trained DRL agent exhibited the following observable decision patterns:

Differentiated Progression Strategy:

The system could distinguish between virtual patient groups with different recovery trajectories and adopt distinct progression paces and training load adjustment strategies. Specifically, for virtual patients classified as “fast recoverers” (top tertile of the healing rate parameter *k*), the agent prescribed a 40% more aggressive mean progression pace compared to “slow recoverers” (bottom tertile), while maintaining comparable safety outcomes. This demonstrates a high potential for data-driven strategy personalization.

Biomarker-Based Anticipatory Decision-Making:

The agent was able to proactively postpone the introduction of high-risk maneuvers in patient models based on subtle abnormal patterns in sEMG signals (e.g., insufficient muscle activation efficiency or temporal discoordination) before significant functional limitations became apparent.

Autonomous Maintenance of Safety Boundaries: During simulated testing, the agent exhibited high sensitivity to pain exacerbation signals (*Δ*VAS > 2), capable of immediately triggering commands for training regression or rest. This validates the effectiveness of the safety weight design in its reward function, ensuring the principle of safety priority in the rehabilitation process.

### 3.2 Comparison of simulated efficacy and safety with static rehabilitation protocols

In a head-to-head comparative test involving 500 virtual patients, the dynamic DRL rehabilitation protocol demonstrated a more balanced advantage in key simulated efficacy and safety indicators.

Efficacy Comparison:

Regarding the key efficiency indicator of “time to safe return to light activity,” the DRL protocol significantly outperformed the conservative static protocol (*P* < 0.05) and was comparable to the aggressive static protocol. However, in terms of the long-term functional score at Week 12 of the simulated rehabilitation cycle, the DRL protocol significantly outperformed both the conservative and aggressive static protocols (*P* < 0.05). This simulation result suggests that the DRL protocol, while pursuing rehabilitation efficiency, may place greater emphasis on long-term recovery quality, avoiding potential damage or recovery plateaus caused by overloading.

Safety Comparison:

The incidence of “re-injury” events triggered by the DRL protocol in the simulation was 2.8%, which was significantly lower than the 7.0% rate observed with the aggressive static protocol (*P* < 0.001). Simultaneously, the number of training interruptions triggered by “pain provocation” during the execution of the DRL protocol was extremely low. These results collectively indicate that the DRL agent, within this simulation environment, successfully achieved a more optimal balance between advancing the rehabilitation process and strictly adhering to safety boundaries.

### 3.3 Performance evaluation of the prognostic prediction module

The TCN prognostic prediction module integrated into the system demonstrated acceptable prediction accuracy and potential clinical early-warning value on an independent test set within the simulation environment.

Prediction Accuracy: The MAEs for predicting functional scores at 2, 4, 8, and 12 weeks into the future, along with comparisons to other models, are detailed in Table 1. The Pearson correlation coefficients (r) between predicted and actual values at all time points were all greater than 0.78. Its prediction performance significantly outperformed the traditional ARIMA model, which served as a baseline, at all time points (*P* < 0.01). To validate the architectural advantages of the TCN, we compared LSTM and GRU models on the same dataset. The results showed that the TCN's MAE for long-term predictions (8-week, 12-week) was reduced by approximately 10%–15% on average, consistent with its theoretical property of more effectively capturing long-range temporal dependencies. The hierarchical receptive field constructed by TCNs through dilated causal convolutions offers the advantages of parallel computation and powerful long-range dependency capture, making them particularly suitable for processing the high-sampling-frequency physiological time-series signals in this study (26, 27). For instance, in a sleep staging task, a multi-scale TCN (MTCNN) achieved an overall classification accuracy of 91.12% by simulating EEG features across different frequency bands, a performance significantly superior to traditional LSTM models (28).

**Table 1:** Comparison of prediction error (MAE) between the TCN model and baseline models at different prediction horizons.

Prediction Horizon	TCN (This Study)	ARIMA	LSTM	GRU
2-week	3.2	5.1	3.6	3.7
4-week	4.8	8.3	5.5	5.6
8-week	6.5	12.1	7.4	7.2
12-week	8.1	15.6	9.5	9.0

All MAE values are measured on the Lysholm centesimal scoring system.

Clinical Early Warning Value: “Delayed recovery” was defined as a simulated Lysholm score at post-operative Week 12 falling below two standard deviations of the age-matched healthy population reference mean. Based on this definition, the prediction module successfully provided an early warning at least two weeks in advance for 82.6% of the cases that ultimately experienced recovery lag. Early identification of recovery trajectory deviation is crucial for timely adjustment of rehabilitation interventions to avoid adverse outcomes. Studies have confirmed that early warning systems based on machine learning models can significantly improve chronic disease management and rehabilitation outcomes. For example, a warning model constructed by integrating indicators such as APTT and lymphocyte count demonstrated significantly superior predictive performance (AUROC=0.971) compared to traditional scoring systems and enabled early warning of infectious complications (29–31). The TCN module in this study, under this simulation setting, demonstrated similar early warning potential, providing a possible prospective basis for dynamically adjusting rehabilitation strategies in clinical practice.

### 3.4 Ablation study and subgroup analysis

To rigorously validate the contribution of individual system components and the personalization capability of the learned policy, we conducted a series of supplementary analyses.

#### 3.4.1 Ablation study: contribution of the TCN prognostic module

To isolate the contribution of the TCN-based prognostic module, we trained a variant agent (denoted as DRL w/o TCN) where the long-term functional gain term (*α* * *Δ*Ŷt + 4w) was removed from the reward function. This agent optimized solely based on immediate functional changes and safety/adherence rewards. When tested on the same 500 virtual patients, the DRL w/o TCN agent achieved a significantly lower 12-week functional score compared to the full DRL agent (mean difference: 8.5 points on the Lysholm scale; *P* < 0.01). Furthermore, the removal of the TCN's foresight led to a relative increase of 21.4% in the simulated re-injury rate (from 2.8% to 3.4%), suggesting that the prognostic module is critical in enabling the agent to forgo short-term progress in favor of long-term safety when necessary.

#### 3.4.2 Subgroup analysis: validation of personalized strategies

To quantitatively validate the agent's personalization capability, we stratified the 500 virtual test patients into three subgroups based on their preset healing rate parameter *k*: “fast” (top 33rd percentile), “medium” (middle 33rd percentile), and “slow” (bottom 33rd percentile) recoverers. Analysis of the prescribed training intensity (continuous action ac) revealed distinct temporal patterns across subgroups. For slow recoverers, the agent prescribed a significantly lower mean intensity during weeks 2–6 post-surgery (mean scaling factor: −0.18 vs. + 0.22 for fast recoverers; *P* < 0.01) and was 3.5 times more likely to issue a “Reduce weight-bearing” discrete action during the same period. Critically, despite these divergent strategies, the 12-week safety outcomes were statistically equivalent across subgroups (re-injury rates: fast, 2.8%; medium, 2.9%; slow, 2.7%; *P* > 0.05 for all pairwise comparisons). This indicates that the agent successfully personalized progression aggressiveness to individual recovery capacity while maintaining a uniform safety standard.

## 4 Discussion

This study preliminarily explored the technical feasibility of a dynamic closed-loop management system for post-operative orthopedic rehabilitation based on PGHD and DRL through simulation modeling. The simulation results indicate that the system can achieve personalized, dynamic adjustment of rehabilitation strategies under idealized conditions and demonstrates potential advantages in simulated safety and long-term functional recovery. The following is a comprehensive discussion of the system's potential clinical significance, innovative value, current limitations, and future translation pathways based on the key findings.

### 4.1 Core findings and the potential innovation of the clinical decision-making paradigm

The core contribution of this study lies in the construction and systematic validation of a DRL agent capable of continuously learning and optimizing rehabilitation strategies in a safe, personalized manner. By integrating multi-modal physiological and behavioral data in real time, it converts abstract clinical goals (such as safety, efficacy, and personalization) into executable, data-driven, continuous decisions. Compared to current rehabilitation models that rely on fixed-periodic assessments and static protocols, this framework demonstrates the potential to transform from a “reactive” intervention to a “prospective” management approach. The agent's ability to differentiate progression strategies among virtual patients—confirmed quantitatively in our subgroup analysis—and its biomarker-based anticipatory decision-making suggest a genuine capacity to identify and respond to subtle differences in individual recovery trajectories. This potential paradigm shift could not only enhance rehabilitation efficiency but also offers a technical framework worth exploring for achieving genuinely precision rehabilitation.

### 4.2 Multi-dimensional innovative value of the system

The innovation of this system is manifested at the methodological, technical integration, and clinical paradigm levels. Methodologically, this study systematically introduces the DRL framework into the context of orthopedic rehabilitation—a domain characterized by continuous decision-making and multiple constraints—and encodes clinical experience into quantifiable optimization objectives through a meticulously designed reward function. Technically, the system achieves deep coupling between the DRL agent and the TCN prognostic prediction module. The TCN module not only provides continuous prediction of future functional recovery but also feeds its predictions back into the DRL's reward function, equipping the system with the prospective computational capability of “making decisions based on predictions.” Our ablation study (Section 2.4.1) provides direct causal evidence for the added value of this technical coupling, demonstrating that the TCN's predictive foresight is essential for achieving the observed balance between efficiency and safety. At the clinical paradigm level, this study constructs a complete digital closed loop of “sensing-decision-prediction-feedback,” providing a scalable architectural template for the development of highly interactive, adaptive Digital Therapeutics (DTx). This closed-loop management paradigm has the potential to enhance patient engagement and provide clinicians with continuous, objective decision support.

### 4.3 Limitations and key challenges for clinical translation

Although the simulation results are encouraging, the fundamental limitations of the current study must be clearly acknowledged, as these are critical obstacles that must be overcome for the system to transition into a real-world clinical setting.

First, the gap between simulation and the real world is the primary and most important limitation. While the simulation environment used in this study integrates biomechanical and tissue healing models, it remains a significant simplification of the complex bio-psycho-social reality of the human body. It cannot fully simulate true individual physiological differences, pain psychology, social support factors, or all potential complication patterns (such as arthrofibrosis or complex regional pain syndrome). The behavioral model of the “virtual patient” in the simulation is idealized, and its results absolutely cannot be directly equated with clinical efficacy; they must be rigorously validated in prospective clinical studies.

Second, model interpretability and safety are core challenges for the clinical acceptance of medical AI. The “black box” nature of DRL models makes their decision-making logic difficult for clinicians to understand directly, which may affect trust and adoption. Future work must integrate Explainable AI (XAI) techniques, such as attention mechanism visualization, *post-hoc* attribution of decisions, or counterfactual analysis, to attempt to elucidate the “clinical logic” behind the system's recommendation of a specific prescription. Research has shown that XAI techniques can significantly enhance the transparency and clinical adoption rate of medical AI systems (32–34). Concurrently, it is imperative to ensure that the system possesses a comprehensive “human-in-the-loop” mechanism and safety monitoring module, enabling clinicians to always retain ultimate decision-making authority and control.

Third, data ethics and engineering implementation face severe challenges. The long-term, continuous collection of PGHD involves patient privacy and security, and the system must strictly comply with data protection regulations such as HIPAA and GDPR. Adopting privacy-preserving computation technologies like federated learning, which allows collaborative model training across multiple centers without centralizing raw data, represents a cutting-edge direction for resolving the contradiction between data silos and privacy (35–37). Furthermore, factors such as device-wearing adherence in home environments, data acquisition quality, and the long-term stable operation of the system are practical issues that need to be addressed during engineering realization.

Finally, the clinical translation pathway must adhere to rigorous evidence-based medicine standards. The translation from simulation to bedside should proceed in phases: initial feasibility studies should focus on system usability, safety, and patient/clinician acceptance; subsequently, well-designed randomized controlled trials (RCTs) should verify clinical efficacy and cost-effectiveness. The design of RCTs for dynamic AI interventions requires special consideration of comparator group settings, outcome measure selection, and the adaptive nature of the intervention, with relevant methodologies continually evolving (38). The ultimate success of the system also depends on seamless integration with existing hospital information systems, electronic health records, and rehabilitation equipment.

### 4.4 Conclusion and future directions

In summary, this proof-of-concept study provides compelling preliminary evidence for the technical feasibility of a DRL-driven, closed-loop system for precision orthopedic rehabilitation. Our framework uniquely couples a wearable sensing layer with a symbiotic DRL-TCN architecture, and through systematic simulation validation—including ablation and subgroup analyses—we demonstrate its potential to safely and dynamically personalize rehabilitation strategies.

Three critical limitations define the path for future work. First, the simulation-reality gap necessitates a progressive clinical validation strategy: beginning with a feasibility study on healthy volunteers, then a pilot safety study on small patient cohorts, before a full-scale randomized controlled trial (RCT). Second, to gain clinical acceptance, we must unpack the “black-box” nature of DRL through *post-hoc* explainability tools such as SHAP analysis on the policy network's state-action mappings. Third, addressing data privacy through approaches like federated learning is not merely a technical requirement but an ethical imperative for ensuring the system's long-term scalability and public trust.

By systematically tackling these challenges, the framework proposed here offers a transformative blueprint for moving beyond static protocols towards genuinely adaptive, data-driven, and patient-centric rehabilitation in sports medicine and beyond.

## Data Availability

The original contributions presented in the study are included in the article/Supplementary Material. further inquiries can be directed to the corresponding author.
